# The effect and mechanism of combination of total paeony glycosides and total ligustici phenolic acids against focal cerebral ischemia

**DOI:** 10.1038/s41598-020-60357-z

**Published:** 2020-02-28

**Authors:** Junfei Gu, Liang Feng, Jie Song, Li Cui, Dan Liu, Liang Ma, Xiaobin Jia

**Affiliations:** 1 School of Chinese Medicine, School of Integrated Chinese and Western Medicine, Nanjing University of Chinese Medicine, Nanjing, Jiangsu 210046 P.R. China; 2 College of pharmacy, China pharmaceutical university, Nanjing, Jiangsu 210023 P.R. China

**Keywords:** Chemical biology, Stroke

## Abstract

The root of *Paeonia lactiflora* Pall. (Chishao, CS) and *Ligusticum chuanxiong* Hort. (Chuanxiong, CX) were widely used as a drug pair in Chinese Medicine, and the combination of CS and CX showed a more significant inhibition on neuronal apoptosis in our previous study. In the present study, total paeony glycosides (TPGs) from CS and total ligustici phenolic acids (TLPAs) from CX were combined to evaluate the synergistic effects against focal cerebral ischemia both *in vitro* and *in vivo*. The combination of TPGs and TLPAs at 7:3 had the best anti-oxidative stress and anti-inflammatory effect on OGD-induced HUVEC. Additionally, the infarction area proportion and neuron apoptosis of rats by TPGs:TLPAs (7:3) was significantly lower than their alone in MCAO rats. Moreover, TPGs: TLPAs of 7:3 showed a more significant effect on decreasing the expression of MMP-2 and MMP-9, and increasing the protein expression or mRNA level of TIMP-1 than other combinations. The optimal ratio of TPGs and TLPAs at 7:3 could bring more remarkable protective effects against focal cerebral ischemia in MCAO rats by alleviating oxidative stress, inflammatory and neuronal apoptosis to protect the blood-brain barrier. Overall, the present study provided benefical evidence for clinical application of CS and CX as a “drug pair”.

## Introduction

As the third highest worldwide cause of mortality^1^, stroke has been treated using antithrombotic therapy^2^, anticoagulant therapy^3,4^, neuroprotective and vascular dilated therapy. Numerous studies have demonstrated that the severity of neuronal apoptosis was associated with neurofunction, indicating a crucial role in the prognosis of ischemic stroke sufferer^5^. Early anti-inflammatory and anti-oxidative stress may promote apoptosis of nerve cells. Thus attenuating these potential damage of nerve cells in ischemic area will be an effective strategy in clinical application.

Chinese medicine (CM) has been used to treat stroke for a long time. Many classical prescriptions for treating cerebral diseases, such as Xiao-xu-ming decoction^6^ are still being commonly used in modern clinial both in China and in other countries^7,8^. *Paeonia lactiflora* Pall. (Chinese name: Chishao, CS) has been used worldwide for centuries and reported to exhibit various pharmacological bioactivities including anticoagulation, cognition enhancement, immunoregulatory^9^ and anticance^10^, antioxidative^11^, and antidiabetic properties^12^. The ischemic stroke protection of *Paeoniae Radix* may be related to its anti-oxidative stress and improvement of brain microcirculation. *Ligusticum chuanxiong* Hort. (Chinese name: Chuanxiong, CX) which belongs to the *Umbelliferae* family, is a valuable medicinal herb used worldwide and has been used for the treatment of atherosclerosis, hypertension, angina pectoris, and anti-inflammatory via acilitating blood circulation and dispersing blood stasis^13^. Our previous study revealed the combination of CS and CX exerted more remarkable protective effects than alone by attenuating ER-stress-dependent apoptotic signaling and protected the blood-brain barrier (BBB)^14^.

Glycosides are the main active constituents in CS, while phenolic acids are the major active components in CX. However, the best effeciacy of these components might be associated with its combination at the optimal ratio. In current study, we use the ischemic model both *in vitro* and *in vivo* to filter the best combination of total paeony glycosides (TPGs) and total ligustici phenolic acids (TLPAs) against focal cerebral ischemia.

## Results

### Cell viability

MTT assay was used to evaluate cell viability for screening the optimal drug concentration of TPGs and TLPAs for Human Umbilical Vein Endothelial Cells (HUVEC) cells. As shown in Table 1, the viability of HUVEC cells ranged from 53.72 ± 2.99% to 101.61 ± 2.11% when treated with different concentration of TPGs from 1 × 10^−3^ g/mL to 1 × 10^−5^ g/mL. While the viability of HUVEC cells ranged from 72.37 ± 5.01% to 99.96 ± 3.25% when treated with TLPAs from 1 × 10^−3^ g/mL to 1 × 10^−5^ g/mL. Thus 2 × 10^−4^ g/mL TPGs with the cell viability of 96.79 ± 1.98% and 2 × 10^−4^ g/mL TLPAs with cell viability of 95.01 ± 2.98% were chosen for further experiments.

**Table 1:** Effect of TPG and TLPA at different concentrations on the survival rates of HUVEC ($\bar{{\rm{x}}}$ ± SD, n = 6).

Group	Dosage (g/mL)	OD	Cell viability (%)
Control blank	—	0.4328 ± 0.1435	100
TPGs	10^−3^	0.2325 ± 0.0247	53.72 ± 2.99
	5 × 10^−4^	0.2765 ± 0.0427	63.88 ± 2.64
	2 × 10^−4^	0.4189 ± 0.0167	96.79 ± 1.98
	10^−4^	0.4203 ± 0.0379	97.12 ± 3.18
	10^−5^	0.4398 ± 0.0622	101.61 ± 2.11
TLPAs	10^−3^	0.3132 ± 0.0482	72.37 ± 5.01
	5 × 10^−4^	0.3658 ± 0.1001	84.51 ± 4.63
	2 × 10^−4^	0.4112 ± 0.0910	95.01 ± 2.98
	10^−4^	0.4179 ± 0.0108	96.57 ± 4.13
	10^−5^	0.4326 ± 0.0240	99.96 ± 3.25

## The effect of TPGs and TLPAs combination against focal cerebral ischemia

### Screening for the optimal ratio *in vitro*

#### Anti-oxidative stress of different ratio with TPGs and TLPAs in OGD-induced HUVEC

As shown in Table 2, comparing with control group, the activities of SOD, NOS, CAT and GSH-Px were significantly decreased in model group (*P* < 0.01), while the levels of MDA, NO, LPO and MPO were significantly increased (*P* < 0.01). Importantly, the activities or levels of SOD, NOS, CAT, GSH-Px, MDA, NO, LPO and MPO were ameliorated after treatment with TPGs, TLPAs and their combination of different ratio. TPGs and TLPAs at the ratio of 8:2 and 7:3 showed the most significantly improvement of on oxidative damage (*P* < 0.01, *P* < 0.05).

**Table 2:** Effect of TPGS and TLPAS on T-SOD, T-NOS, i-NOS, CAT, LPO, MPO and GSH-PX activity and MDA, NO content in OGD-induced HUVEC ($\bar{{\rm{x}}}$ ± SD, n = 3).

Group		T-SOD (U/mL)	MDA (nmol/mL)	NO (μmol/L)	T-NOS (U/mL)	i-NOS (U/mL)	CAT (U/mL)	LPO (μmol/L)	MPO (U/L)	GSH-PX (μmol/L)
Control		4.55 ± 0.08	0.52 ± 0.07	200.56 ± 3.29	3775.56 ± 21.42	3131.64 ± 22.72	0.86 ± 0.05	0.13 ± 0.02	1145.13 ± 133.83	76.75 ± 1.28
Model		1.23 ± 0.24^##^	1.73 ± 0.09^##^	509.16 ± 1.11^#^	1731.38 ± 108.14^##^	1506.16 ± 72.15^##^	0.49 ± 0.05^#^	0.32 ± 0.08^##^	3692.33 ± 194.27^##^	20.84 ± 2.59^##^
Ni		3.79 ± 0.24^**^	0.76 ± 0.03^**^	326.08 ± 7.73^**^	2989.82 ± 130.77^**^	2197.70 ± 52.66^*^	0.67 ± 0.05^*^	0.25 ± 0.04^*^	1859.59 ± 107.16^**^	48.16 ± 1.97^**^
TPGs: TLPAs	TPGs	2.48 ± 0.10	1.27 ± 0.03^*^	454.02 ± 7.10	1897.91 ± 110.30	1581.25 ± 22.62	0.54 ± 0.07	0.29 ± 0.08^*^	3554.28 ± 144.68	29.61 ± 0.73
	9:1	3.86 ± 0.14^*^	1.21 ± 0.03^*^	448.36 ± 6.59	1938.38 ± 121.16	1650.76 ± 116.92	0.63 ± 0.09^*^	0.31 ± 0.10	3109.44 ± 163.32	37.48 ± 0.90^*^
	8:2	4.02 ± 0.20^*^	1.05 ± 0.02^**^	421.01 ± 7.17^**^	2176.66 ± 174.60^*^	1652.38 ± 85.40	0.64 ± 0.03^*^	0.30 ± 0.04	3272.25 ± 109.90	37.81 ± 0.57^*^
	7:3	4.25 ± 0.31^**^	0.62 ± 0.19^**^	425.09 ± 12.26^**^	2182.19 ± 119.39^*^	1974.41 ± 78.21^*^	0.67 ± 0.07^*^	0.28 ± 0.07^*^	2402.06 ± 143.20^**^	44.01 ± 5.04^**^
	6:4	3.68 ± 0.25^*^	0.72 ± 0.06^**^	435.94 ± 10.05^*^	1993.05 ± 185.68^*^	1818.85 ± 68.42^*^	0.65 ± 0.06^*^	0.28 ± 0.06^*^	2333.63 ± 140.28^**^	42.71 ± 4.08^**^
	5:5	4.13 ± 0.19^**^	1.09 ± 0.06^**^	440.94 ± 15.46^*^	1940.21 ± 124.37^*^	1790.89 ± 101.54^*^	0.53 ± 0.02	0.30 ± 0.05	3420.65 ± 111.45	39.42 ± 1.21^*^
	4:6	3.72 ± 0.32^*^	1.29 ± 0.10^*^	459.68 ± 14.39^*^	1860.21 ± 105.21	1615.82 ± 84.42	0.64 ± 0.08^*^	0.30 ± 0.07	3542.78 ± 107.81	39.33 ± 1.07^*^
	3:7	2.96 ± 0.12^*^	1.68 ± 0.27	405.14 ± 14.39^**^	1849.35 ± 107.41	1696.61 ± 65.26	0.59 ± 0.02^*^	0.30 ± 0.05	3507.08 ± 166.56	32.65 ± 1.93^*^
	2:8	3.22 ± 0.16^*^	1.12 ± 0.12^*^	451.31 ± 5.05^*^	1839.71 ± 55.27	1641.02 ± 65.26	0.57 ± 0.07	0.31 ± 0.05	3610.03 ± 190.00	28.58 ± 0.97
	1:9	1.55 ± 0.86	1.41 ± 0.01	484.59 ± 4.30	1861.80 ± 57.53	1663.29 ± 28.37	0.52 ± 0.03	0.32 ± 0.02	3373.75 ± 173.55	27.64 ± 0.75
	TLPAs	2.20 ± 0.25	1.56 ± 0.37	470.84 ± 1.98^*^	1790.10 ± 65.52	1672.32 ± 109.39	0.57 ± 0.05	0.32 ± 0.08	3532.15 ± 178.17	27.06 ± 0.44

Notice: ^##^P < 0.01 or ^#^P < 0.05, compared with control group; **P < 0.01 or *P < 0.05, compared with model group.

#### Anti-inflammatory of different ratio with TPGs and TLPAs in OGD-induced HUVEC

As depicted in Table 3, the contents of TNF-α, IL-6 and sICAM-1 were significantly increased after modeling (*P* < 0.01, *P* < 0.05). The contents of TNF-α, IL-6 and sICAM-1 were all decreased in different degrees after treatment of TPGs, TLPAs and their combination of different ratio. A more significant decrease of these inflammatory cytokines was observed in HUVEC treated with TPGs and TLPAs combination at the ratio of 7:3 (*P* < 0.01, *P* < 0.05).

**Table 3:** Effect of XS on TNF-α, IL-6, sICAM-1expression in OGD-induced HUVEC ($\bar{{\rm{x}}}$ ± SD, n = 3).

Group		TNF-α (ng/L)	IL-6 (ng/L)	sICAM-1 (ng/L)
Control		21.57 ± 2.38	10.30 ± 1.24	77.51 ± 6.98
Model		27.51 ± 1.91^##^	28.00 ± 0.26^#^	105.16 ± 7.91^##^
Ni		24.63 ± 3.38^*^	9.46 ± 0.36^**^	77.44 ± 6.72^**^
TPGs:TLPAs	G	26.49 ± 2.17	22.02 ± 1.37^*^	99.22 ± 7.69
	9:1	25.66 ± 1.74	19.19 ± 0.16^*^	97.15 ± 6.31
	8:2	25.62 ± 2.37	19.05 ± 0.27^**^	89.18 ± 7.87^**^
	7:3	24.26 ± 3.28^**^	10.98 ± 1.17^**^	87.28 ± 9.79^**^
	6:4	24.44 ± 2.14^**^	18.35 ± 0.27^*^	92.84 ± 8.67^*^
	5:5	24.47 ± 3.28^*^	18.68 ± 0.26^*^	94.79 ± 9.63^*^
	4:6	24.75 ± 1.65^*^	19.21 ± 0.16^*^	95.79 ± 3.69^*^
	3:7	25.13 ± 1.48^*^	19.98 ± 0.17	97.21 ± 5.80
	2:8	25.27 ± 1.35	21.08 ± 1.07	101.71 ± 7.14
	1:9	26.02 ± 1.95	24.06 ± 0.27	103.79 ± 2.02
	F	27.13 ± 1.02	24.70 ± 0.20	103.79 ± 3.70

Notice: ^##^P < 0.01 or ^#^P < 0.05, compared with control group; **P < 0.01 or *P < 0.05, compared with model group.

### The effect of TPGs and TLPAs at the best optimal ratio on MCAO rats

#### Improvement of TPGs:TLPAs at 7:3 on neurological deficit score of MCAO rats

As the results showed in Table 4, at 24 h after cerebral ischemia, the neurological deficit score of the sham-operated rats was 0, indicating that the sham-operated rats did not have neurological impairment symptoms. Compared with the sham-operated group, the neurological deficit scores in the model group were significantly increased. (P < 0.01). After administration, the score of neurological deficit in rats was significantly lower (P < 0.01, P < 0.05), and the changes were with the prolongation of administration time. TPGs:TLPAs at the ratio of 7:3 showed the best protective effect on the neurological deficit.

**Table 4:** The neurological function of rats after MCAO ($\bar{{\rm{x}}}$ ± SD, n = 24).

Groups	24 h	1 week	2 week
Sham	0	0	0
Model	3.32 ± 0.09^##^	3.35 ± 0.13^##^	3.34 ± 0.13^##^
Ni	3.30 ± 0.17^##^	2.23 ± 0.21^**@@^	2.08 ± 0.17^**@^
TPGs	3.29 ± 0.22^##^	2.62 ± 0.20^*@^	2.30 ± 0.20^**@@^
TPGs:TLPAs (7:3)	3.30 ± 0.18^##^	2.48 ± 0.13^*$&@@%^	2.21 ± 0.22^**$$&@%^
TPGs:TLPAs (5:5)	3.28 ± 0.23^##^	2.61 ± 0.06^*@@^	2.29 ± 0.19^**$$@^
TLPAs	3.31 ± 0.19^##^	2.87 ± 0.13^*@^	2.69 ± 0.11^*^

Notice: ^##^P < 0.01, compared with sham group; **P < 0.01 or *P < 0.05, compared with model group; ^$$^P < 0.01 or ^$^P < 0.05, compared with TLPAs group; ^&^P < 0.05, compared with TPGs group; %P < 0.05, compared with TPGs:TLPAs (5:5) group; ^@@^P < 0.01 or ^@^P < 0.05, compared with the previous group.

#### Improvement of TPGs:TLPAs at 7:3 on infarction area of MCAO rats

**Figure 1:**
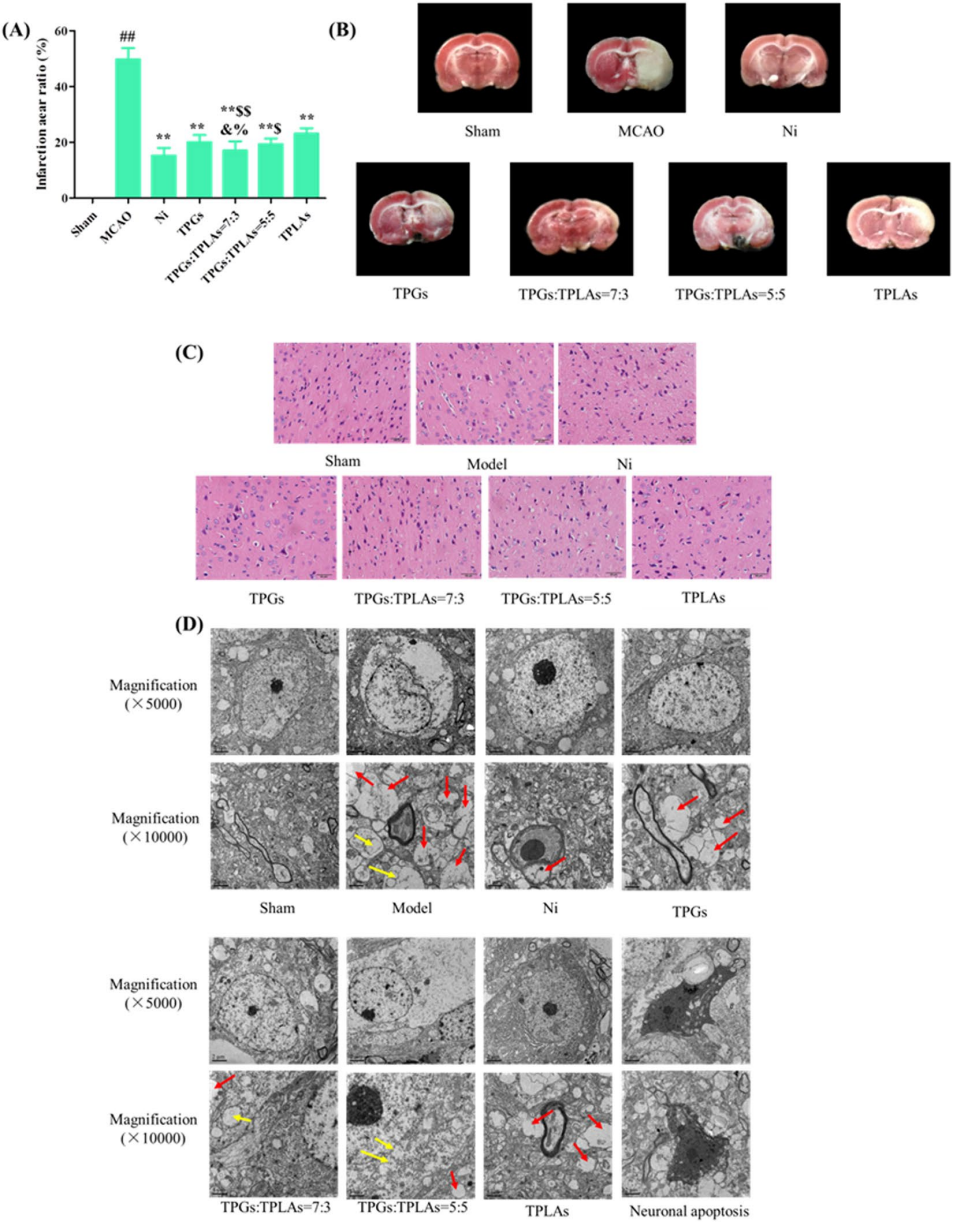
Infarction changes of different treatment rats after cerebral ischemia. (**A**) Images of brain by TTC staining; (**B**) Infarction aera ratio of different treatment rats after cerebral ischemia; (**C**) Histopathological changes of cerebral ischemia after treatment; (**D**) Effect of XS on the neuronal intracellular environment of rat hippocampus neurons after cerebral ischemia. a, synaptic myelin disappearance (red arrows); b, swelling (yellow arrows).

As shown in Fig. 1A, infarction areas were shown in white. There was no infarction in the sham operation group, while infarction occurred in each group after the model was established. The cerebral infarct size ratio of the model group was reached 49.72%, which was significantly different from the sham operation group (P < 0.01). Compared with the model group, the infarct size of cerebral ischemia rats was significantly reduced after administration (P < 0.01, Fig. 1B). When TPGs:TLPAs at the ratio of 7:3, the treatment of infarct size in cerebral ischemia rats was most effective, showed a significantly lower in infarction areas than other combinations (P < 0.01, P < 0.05). There was no difference in cerebral infarct size ratio between TPGs:TLPAs treatment at 5:5 and TPG treatment alone, but the effect was better than that of the TLPAs alone group (P < 0.05).

#### Anti-oxidative stress activity of TPGs:TLPAs at 7:3 in MCAO rats

Anti-oxidative stress activity of TPGs:TLPAs combinations in MCAO rats was presented in Table 5. Comparing with sham control group, the activities of SOD, CAT and GSH-Px of model rats were significantly decreased (*P* < 0.01, *P* < 0.05), while the levels of MDA, and LPO were significantly increased (*P* < 0.01, *P* < 0.05). The activities of SOD, CAT, GSH-Px, MDA, and LPO were significantly reversed by the treatment with Ni, TPGs, TLPAs, TPGs:TLPAs at 7:3 and TPGs:TLPAs at 5:5. Importantly, TPGs:TLPAs at 7:3 ratio has a more significant amelioration on these activities or levels, compared with other combinations (*P* < 0.01, *P* < 0.05).

**Table 5:** The effect of XS on SOD, CAT, GSH-Px activity and LOP, MDA content in rats serum of different treatment groups after cerebral ischemia ($\bar{{\rm{x}}}$ ± SD, n = 24).

Groups	SOD (U/mL)	CAT (U/mL)	GSH (μmol/L)	MDA (U/mL)	LPO (μmol/L)
Sham	198.68 ± 2.26	12.98 ± 1.01	410.23 ± 19.17	38.12 ± 8.71	5.09 ± 0.73
Model	121.33 ± 5.40^##^	5.06 ± 1.30^##^	370.11 ± 23.91^#^	60.22 ± 13.56^##^	14.16 ± 1.97^##^
Ni	191.36 ± 14.30^**^	10.11 ± 1.82^**^	397.16 ± 13.43^**^	41.11 ± 8.76^**^	5.28 ± 0.38^**^
TPGs	179.35 ± 4.97^**^	8.31 ± 0.87^*^	389.67 ± 10.91^*^	50.69 ± 12.65^*^	6.82 ± 1.75^**^
TPGs:TLPAs (7:3)	189.56 ± 2.66^**$$&&**%**^	10.12 ± 1.29^**$$&&**%**^	395.37 ± 19.38^**$&^	43.77 ± 7.81^**$&&**%**^	5.35 ± 0.68^**$$&**%**^
TPGs:TLPAs (5:5)	180.72 ± 3.69^**$&&^	9.01 ± 1.15^**$$&^	391.56 ± 14.69^**$&^	48.65 ± 10.03^*$&^	6.17 ± 0.94^**$$^
TLPAs	173.18 ± 14.30^**^	8.39 ± 1.29^**^	389.15 ± 19.38^*^	50.36 ± 7.81^*^	8.05 ± 0.68^*^

Notice: ^##^P < 0.01 or ^#^P < 0.05, compared with sham group; **P < 0.01 or *P < 0.05, compared with model group; ^$$^P < 0.01 or ^$^P < 0.05, compared with TLPAs group; ^&&^P < 0.01 or ^&^P < 0.05, compared with TPGs group; ^%%^P < 0.01 or ^%^P < 0.05, compared with TPGs:TLPAs (5:5) group.

#### Effects of TPGs:TLPAs at 7:3 on functional recovery and anti-apoptosis of MCAO rats

**Figure 2:**
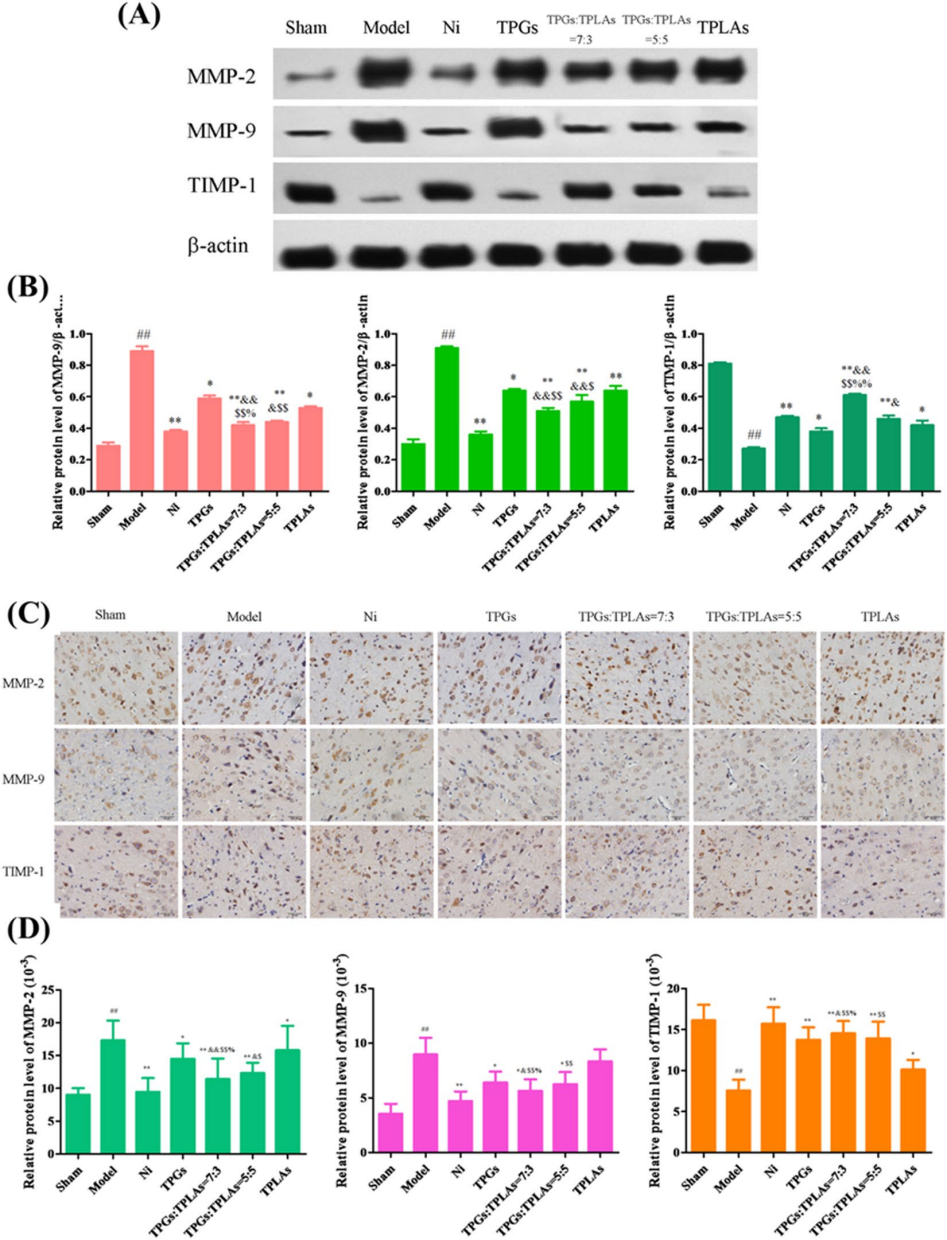
Effect of TPGs and TLPAs on MMP-2, MMP-9 and TIMP-1 protein expression in cerebral ischemia rats. (**A**) Images of western blot; (**B**) Relative protein expression of MMP-2, MMP-9 and TIMP-1; (**C**) Images of immunohistochemical staining; (**D**) Relative protein expression of MMP-2, MMP-9 and TIMP-1. ^##^*P* < 0.01, compared with sham group; ^**^P < 0.01 or ^*^P < 0.05, compared with model group; ^$$^*P* < 0.01 or ^$^*P* < 0.05, compared with TPGs group; ^&&^*P* < 0.01 or ^&^*P* < 0.05, compared with TLPAs group; ^%^*P* < 0.05, compared with TPGs^:^TLPAs (5:5) group.

Clear and integral tissue structure was observed in sham group with clear neuronal nuclei, relatively intact nuclear membrane and normal glial cells and capillary morphogenesis (Fig. 2C). However, in model group, edema of the neuropile were observed in model group, and most neurons in the damage areas appeared shrunken with eosinophilic cytoplasm and triangulated pyknotic nuclei. Histopathological abnormalities were significantly improved by TPGs and TLPAs combinations. Particularly, the treatment of TPGs:TLPAs (7:3) had the most obvious effect on improving the ischemic injury compared with other groups (*P* < 0.01, *P* < 0.05).

In sham control group, polygons neurons, abundant cytoplasm, clear nucleoli and integral cell membrane were observed by TEM (Fig. 1D) by TEM. After MCAO modeling, neurons were swelled, organelles were broken or disappeared, and cell membranes were adhered with synapses. Meanwhile myelin in most synapses from surrounding stroma was disappeared (red arrow) or swelled (yellow arrow). After being treated with Ni, TPGs, TLPAs, TPGs:TLPAs at 7:3 and 5:5, apoptotic neurons were significantly ameliorated. Especially, the treatment with TPGs:TLPAs (7:3) had the most obvious improvement on neurons apoptosis compared with other combinations (*P* < 0.01, *P* < 0.05).

#### Regulation of TPGs:TLPAs at 7:3 on MMP-9, PA, and PAI-1 in MCAO rats

As shown in Table 6, comparing with sham control group, the activities of MMP-9 and PAI-1 were significantly decreased in model group (*P* < 0.01), whereas the activities of PA were significantly increased (*P* < 0.01). After intervening by Ni, TPGs, TLPAs, TPGs:TLPAs at 7:3 and 5:5, the activities of MMP-9 and PAI-1 were significantly increased, whereas the activities of PA was significantly decreased. TPGs and TLPAs at the ratio of 7:3 significantly changed the activity to a higher level when compared with other combinations (*P* < 0.01, *P* < 0.05).

**Table 6:** MMP-9, PA, PAI-1 content in different treatment rats serum after MCAO ($\bar{{\rm{x}}}$ ± SD, n = 24).

Groups	MMP-9 (ng/mL)	PA (mg/mL)	PAI-1 (ng/mL)
Sham	38.12 ± 1.87	241.91 ± 26.19	51.13 ± 9.71
Model	60.03 ± 1.91^##^	67.36 ± 18.01^**##**^	185.83 ± 10.12^**##**^
Ni	42.05 ± 1.33^**^	221.46 ± 15.43^**^	69.27 ± 9.31^**^
TPGs	50.43 ± 1.51^*^	191.52 ± 16.51^**^	107.42 ± 10.19^*^
TPGs:TLPAs (7:3)	45.33 ± 2.01^**&$%^	219.93 ± 19.06^**&$%^	95.37 ± 8.76^**&&$$%^
TPGs:TLPAs (5:5)	48.62 ± 1.88^*$^	201.83 ± 20.31^**$$^	103.13 ± 13.51^**&^
TLPAs	50.43 ± 1.92^*^	190.72 ± 17.31^**^	110.19 ± 10.03^*^

Notice: ^##^P < 0.01 or ^#^P < 0.05, compared with sham group; **P < 0.01 or *P < 0.05, compared with model group; ^$$^P < 0.01 or ^$^P < 0.05, compared with TLPAs group; ^&&^P < 0.01 or ^&^P < 0.05, compared with TPGs group; ^%%^P < 0.01 or ^%^P < 0.05, compared with TPGs:TLPAs (5:5) group.

#### Protection of TPGs:TLPAs at 7:3 on blood-brain barrier in MCAO rats

Described as shown in Fig. 2, protein expression levels of MMP-2 and MMP-9 in rats brain of model group were significantly raised while TIMP-1 protein expression was significantly reduced after being compared with sham control group (*P* < 0.05). However, protein expression of MMP-2 and MMP-9 were significantly decreased whereas TIMP-1 increased by Ni, TPGs, TLPAs, TPGs:TLPAs at 7:3 and 5:5 (*P* < 0.01, *P* < 0.05). Of note that TPGs:TLPAs (7:3) improved protein expressions of MMP-2, MMP-9 and TIMP-1 far more significantly than other groups (*P* < 0.01, *P* < 0.05).

#### Protection of TPGs:TLPAs at 7:3 on apoptotic protein in MCAO rats

**Figure 3:**
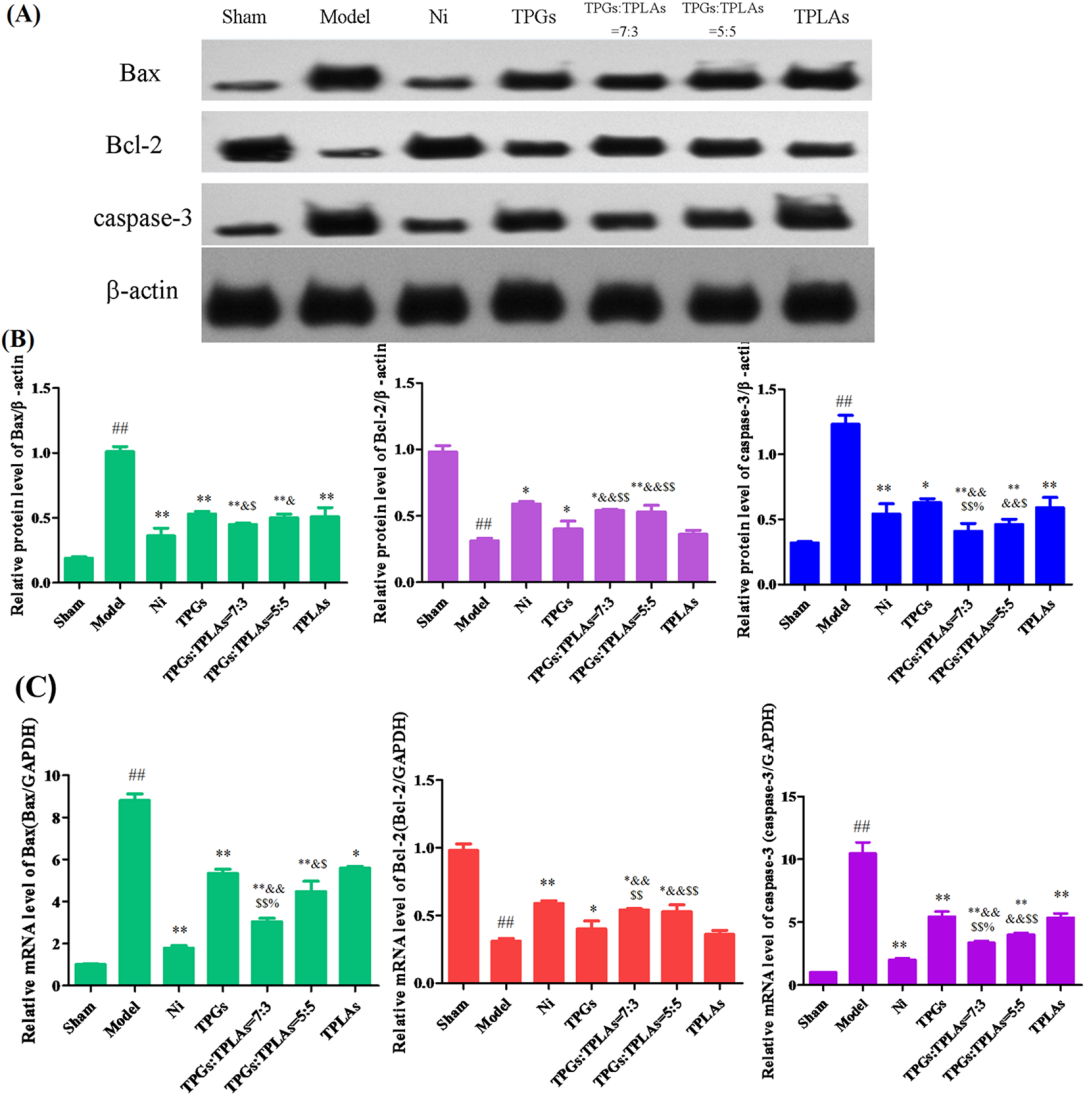
Effect of TPGs and TLPAs on Bax, Bcl-2 and caspase-3 protein expression in cerebral ischemia rats. (**A**) Images of western blot; (**B**) Relative protein expression of Bax, Bcl-2 and caspase-3; (**C**) The mRNA level in cerebral ischemia rats. ^##^*P* < 0.01, compared with sham group; ^**^P < 0.01 or ^*^P < 0.05, compared with model group; ^$$^*P* < 0.01 or ^$^*P* < 0.05, compared with TPGs group; ^&&^*P* < 0.01 or ^&^*P* < 0.05, compared with TLPAs group; ^%^*P* < 0.05, compared with TPGs^:^TLPAs (5:5) group.

After modeling, protein expression of Bax and caspase-3 were significantly enhanced whereas Bcl-2 was apparently declined (*P* < 0.01). Compared with model group, protein expressions of Bax and caspase-3 in rats brain decreased significantly while Bcl-2 was significantly increased after treatment the with Ni, TPGs, TLPAs, TPGs:TLPAs at 5:5 and 7:3 for two weeks (*P* < 0.01, *P* < 0.05) (Fig. 3A). It was important that protection of TPGs:TLPAs at 7:3 on apoptosis in MCAO rats was significantly better than any other groups (*P* < 0.01, *P* < 0.05).

Compared with sham control group, mRNA level of Bax and caspase-3 in rats brain were apparently enhanced while mRNA level of Bcl-2 was apparently declined (*P* < 0.01) in model group. The treatment of Ni, TPGs, TLPAs, TPGs:TLPAs (5:5) and TPGs:TLPAs (7:3) significantly decreased the mRNA levels of Bax and caspase-3 whereas apparently increased Bcl-2 mRNA level when compared with model group (*P* < 0.01, *P* < 0.05) (Fig. 3B). This protection of TPGs and TLPAs at the ratio of 7:3 on apoptosis in MCAO rats was significantly better than other groups (*P* < 0.01, *P* < 0.05). The results of both Western blotting and qPCR are consistent.

**Figure 4:**
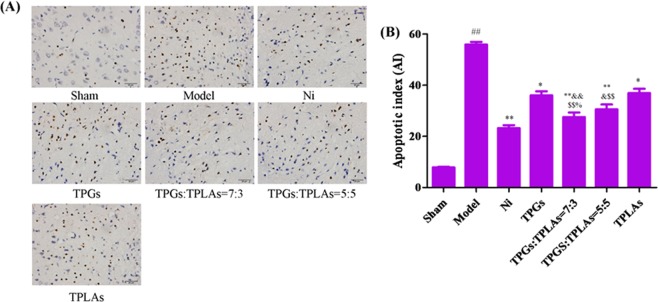
Effect of TPGs and TLPAs on TUNEL staining in brain tissue of cerebral ischemia rats. (**A**) Images of TUNEL staining; (**B**) Apoptotic index. ^##^*P* < 0.01, compared with sham group; ^**^P < 0.01 or ^*^P < 0.05, compared with model group; ^$$^*P* < 0.01 or ^$^*P* < 0.05, compared with TPGs group; ^&&^*P* < 0.01 or ^&^*P* < 0.05, compared with TLPAs group; ^%^*P* < 0.05, compared with TPGs:TLPAs (5:5) group.

In model group, apoptotic cells were increased with apoptotic index (AI) significantly enhanced compared with sham control group (*P* < 0.01). After treatment with Ni, TPGs, TLPAs, TPGs:TLPAs (5:5) and TPGs:TLPAs (7:3) for two weeks, AI of rats’ brain were significantly declined (*P* < 0.01, *P* < 0.05) (Fig. 4). It was worth pointing out that 7:3 ratio was more reasonbale for anti-apoptosis in MCAO rats than other groups (*P* < 0.01, *P* < 0.05).

## Discussion

TCM, consisting of mutiple herbs, was benefical to synergistic effect of multi-components for the treatment and prevention of diseases. In traditional application, CS (*Paeonia lactiflora* Pall.) and CX (*Ligusticum chuanxiong* Hort.) were generally used as “drug pair” to treat the stasis and stroke. As known, CS was used for “eliminating blood stasis and relieving pain” (Chinese Pharmacopoeia 2015). Modern phytochemical and pharmacological studies have shown that monoterpene glycosides, galloyl glucoses and phenolic compounds are the major bioactive components of CS. Research showed its major compound paeoniflorin was benefical for auto-inflammatory and autoimmune disease treatment^9^. In this study, organic acids including ferulic acid and caffeic acid were isolated and identified from *Ligusticum Chuanxiong* Hort. These components have been proved to hold chemoprevention on apoptosis and reduction on the risk of ROS-mediated diseases^15^. According to our previously study, the combination of CS and CX have potential effects on curing cerebral disease or stroke. However, the best effeciacy of these components might be associated with its combination with the optimal ratio. Total paeony glycosides (TPGs) are the main active constituents in CS and total ligustici phenolic acids (TLPAs) are the major active components in CX. TPGs were reported to have a good effect on both cerebral ischemia and cardiac ischemia^16,17^.

In current study, we use the ischemic model both *in vitro* and *in vivo* to identify the best combined ratio of TPGs and TLPAs against focal cerebral ischemia. The optimal ratio of TPGs and TLPAs at 7:3 could bring more remarkable protective effects against focal cerebral ischemia in MCAO rats by alleviating oxidative stress, inflammatory and neuronal apoptosis to protect the blood-brain barrier. The present study provided benefical evidence for clinical application of CS and CX as a “drug pair”.

Cerebral disease or stroke is caused by lack of oxygen to the cerebrum, however their pathophysiology are still not completely clear^1^. Cerebral ischemia, with risk factors including hypertension, diabetes, hyperlipidemia, smoking and tiredness, may cause irreversible structural changes in a specific vascular territory^18^. However, the effect of cerebral ischemia on BBB, a diffusion barrier essential for maintenance of homeostasis and normal function of the central nervous system, has been broadly investigated^10^. In current study, we investigated the protection of the combination of TPG and TLPA on BBB by anti-inflammation, anti-oxidative stress and anti-apoptosis effects.

Because of complicated factors for cerebral disease or stroke, it is inevitably to be a comprehensive pathological process. Cerebral inflammation participated in all stages of ischemic cascade which plays a crucial role in the pathophysiology of ischemic stroke^19^, while oxidative stress has also been reported to be one of the main cause in ischemia reperfusion injury^20^.

Matrix metalloproteinases (MMPs), a family of zinc-binding proteolytic enzymes, contribute to the pathology of cerebral infarction by degrading a number of extracellular matrix molecules to breakdown the blood-brain barrier^21^ MMPs-mediated BBB disruption has been found to be a critical pathological mechanism of cerebral disease or stroke^22^. However, this activation of MMPs is complexly modulated by transcriptional regulators and inhibitor tissue inhibitor of metalloproteinases (TIMPs)^23^. Under normal physiological conditions, MMPs are directly responsible for pathogenesis of cerebrovascular diseases and their activity is regulated by TIMPs^24^. The evidence showed that MMP-2 and MMP-9 might be up-regulated and activated during cerebral disease or stroke which are expressed particularly frequently in the nervous system^25^. MMP-2 also plays a significant role in the regulation of activity of platelets^26^, while MMP-9 damages matrix components of the basement membrane leading to neuro-inflammation after focal cerebral ischemia by maintaining the integrity of cerebral vasculature^27^. MMP-9, one of the most important MMPs correlated with BBB damage, accompanied with its endogenous inhibitor TIMP-1, has been confirmed to be important in vascular remodeling and neuro-protective effect^28^. This study indicated that there was a more significant effect of TPGs:TLPAs (7:3) on adjusting MMP-2, MMP-9 and TIMP-1 protein expression and mRNA level than other groups.

It has been proved that apoptosis is associated with cerebral disease or stroke leading to BBB dysfunction, inflammation and oxidative stress resulting in brain damage^29–31^. Histopathological changes and TEM images in current study indicated that TPGs and TLPAs especially the TPGs:TLPAs at 7:3 could alleviate neuronal apoptosis.

## Conclusions

In this study, the optimal ratio of TPGs and TLPAs on improving cerebral ischemia damage was compared and screened according to ODG-induced HUVEC cells *in vitro* and MCAO rats *in vivo*. The results demonstrated the combination TPGs and TLPAs at 7:3 might be more benetifical to this protective effect via alleviating oxidative stress, inflammation and neuronal apoptosis. This study provided an evidence of the combination of CS and CX for clinical application as “drug pair”, and more importantly, the study revealed that the proper ratio of TPGs and TLPAs could be critical for CS-CX “drug pair” in curing focal cerebral ischemia.

## Materials and Methods

### Materials and chemicals

*Paeonia lactiflora* Pall. (CS) was offered by Sichuan new Lotus Chinese Herbal Medicine Co., Ltd. (Chengdu, China) with the lot number 1311060. *Ligusticum chuanxiong* Hort. (CX) was got from Songling Chinese Herbal Medicine Co., Ltd. (Nanjing, China) with the lot number 131017. The pharmaceutical botanies of the medicinal material were identified by Prof. Dekang Wu from Nanjing University of Chinese Medicine (Nanjing, China).

Nimodipine (Ni, approval number was H20003010), as a positive control drug, was obtained from Bayer HealthCare (Beijing, China). Kits for SOD, MDA, CAT, GSH-Px and LPO assay were purchased from Jiancheng Bioengineering Institute (Nanjing, China). ELISA kits for MMP-9, PA and PAI-1 were provided by Beyotime Biotech. Co., LTD. (Shanghai, China). Hematoxylin-eosin staining solution was offered by Shanghai Yuanmu Biotechnology Co., Ltd. (Shanghai, China). RPMI 1640 medium and trypsin were provided by KeyGEN Biotech Co., Ltd (Nanjing, China). Fetal bovine serum (FBS) was purchased from Gibco/BRL (Grand Island, USA). 3–4, 5-dimethyl-2-thiazolyl)-2, 5-diphenyl-2-H-tetrazolium bromide (MTT) and dimethyl sulfoxide (DMSO) were provided by Sigma (St. Louis, USA). Rabbit anti-mouse Bax, Bcl-2, and TIMP-1 monoclonal antibodies were offered by Santa Cruz Biotechnology, Inc. (Dallas, USA). Rabbit anti-mouse MMP-9, PERK, XBP-1, ATF-6 and CHOP monoclonal antibodies were obtained from Abcam, Inc. (Cambridge, UK). Other rabbit anti-mouse monoclonal antibodies were got from Boster Biological Engineering Co., Ltd. (Wuhan, China). EliVision plus and DAB kits were offered by MXB Biological Technology Co., Ltd. (Fuzhou, China).

### Preparation of TPGs and TLPAs

The preparation of TPGs was performed as previous studies with modifications. Pieces of CS with 1 kg were weighed and extracted twice (1.5 h/time) with 10 times amount of 70% (v/v) ethanol in reflux extraction device. The extraction was concentrated to 1 g decoction pieces/mL. TPGs was defined as the 30% ethanol eluant chromatographed with a D-101 macroreticular resin (Cangzhou Bon Adsorber Technology Co., Ltd, Cangzhou, China). On the basis of previous studies^32^, 1 kg CX were weighed and extracted twice (1.5 h/time) with 15 times amount of 80% (v/v) ethanol in reflux extraction device. The extraction was concentrated to 1 g decoction pieces/mL and was chromatographed with AB-8 macroreticular resin (Cangzhou Bon Adsorber Technology Co., Ltd, Cangzhou, China) column. Finally, the eluant of 30% ethanol was defined as TLPAs. The yield of CS and CX extraction was 17.81% and 16.63%, respectively. The content of TPGs was 3.62% after extraction with 70% ethanol. And after purification by macroporous resin, the content of the TPGs used in the study was 47.92% finally. While the content of TLPAs was 2.42% after extraction with 80% ethanol, and the content of the TLPAs used in the study was 41.43% finally after purification(The data shows in Supplementary Information).

### Cells culture

HUVEC was purchased from Shanghai Institute of Biochemistry and Cell Biology (Shanghai, China) and cultured in an incubator with 5% CO_2_ at 37 °C. Cells were treated with RPMI 1640 medium containing 80 units/mL of penicillin, 0.08 mg/mL of streptomycin (KeyGEN, China) and 10% fetal bovine serum (FBS, Gibico, USA).

### Cell proliferation assay by MTT

After 90% confluence, cells were digested by 0.25% trypsin-0.02% EDTA for the passage. A cell density of 5000 cells/well was seeded into 96-well plates (100 μL). Cells were treated with different concentrations of TPGs prepared (10^−3^, 5 × 10^−4^, 2 × 10^−4^ and 10^−5^ g/mL) and TLPAs (10^−3^, 5 × 10^−4^, 2 × 10^−4^ and 10^−5^ g/mL) for 24 h respectively. MTT stock solution of 5.0 mg/mL (10 μL) was added to each well for 4 h to form water-insoluble purple crystal formazan. At the end of incubation, DMSO of 150 μL was added for 10 min microvibration after removing medium. The absorbance was measured at 570 nm on a microplate reader (Thermo, NewYork, USA).

### Cell OGD model and drug treatment

HUVECs modeled with oxygen glucose deprivation (OGD) was used as an ischemic model *in vitro* in according to previous reports. Cells of 90% confluence were cultured in 96-well plates (5000 cells/well, 100 μL). For simulated ischemia-reperfusion, cells were placed in a hypoxic incubator (5% CO_2_, 95% N_2_) at 37 °C for 4 h.

Then serum-free RPMI 1640 medium was used in control group and model group. The other OGD cells were divided into 12 groups (n = 6/group) including positive control group (Nimodipine 10 μM); TPGs group; TLPAs group; TPGS:TLPAs at 9:1, 8:2, 7:3, 6:4, 5:5, 4:6, 3:7, 2:8 and 1:9 ratios. The treatment dosage was 2 × 10^−4^ g/mL.

### Animal model and drug treatment

Male Sprague-Dawley rats (280 ± 20 g, licence number: SCXK (Su) 2015–0001) were supplied by Nantong University (Nantong, China). Rats were kept at a temperature of 25 °C and the relative humidity of 45%. All the experiment procedures were approved by the Institutional Animal Care and Use Committee of the Jiangsu Provincial Academy of Chinese Medicine in accordance with published National Institutes of Health guidelines, the ethics code number of our animal experimental was ZYY20151207. Before modeling, rats were housed for 7 d to adapt to the environment on a 12 h/12 h light/dark cycle with food and water *ad libitum*.

The middle cerebral artery occlusion (MCAO) model was performed as described previously. Rats were injected intraperitoneally 10% chloral hydrate (300 mg/kg) for anesthetizing. After anesthetized, rats were laid in dorsal recumbency and the right side of carotid artery was exposed and isolated. The origin of the right middle cerebral artery (MCA) was occluded by inserting a 0.26 mm heparin-dampened monofilament nylon suture (Ethicon, Inc., Osaka, Japan) with a heat-rounded tip from the external carotid artery (ECA) to the right internal carotid artery (ICA). Thread at the distal end of common carotid artery (CCA) was fastened when the suture was advanced closed to the origin of the MCA. Extravascular suture was cut before the closed neck incision. After preventing wound infection, rats in the sham group were performed with the same surgical procedures without insertion of the heparin-dampened monofilament nylon suture. The body temperature of these rats was maintained at 37 °C during surgery. After surgery, sodium penicillin (100,000 U/a rat/d) was given to prevent infection for three days successively.

Neurological examination was conducted to screen the successful models after modeling for 24 h. The successfully modeled rats were randomly divided into 7 groups (n = 24/group): model group (MCAO, Saline 10 mL/kg); sham control group (Saline 10 mL/kg); positive control group (Nimodipine 7.53 mg/kg); TPGs group; TLPAs group; TPGs:TLPAs at 7:3 group and TPGs:TLPAs at 5:5 group. The treatment drug dose was 500 mg/kg according to the previous research^17^. Equivalent saline was given to the sham rats and model rats. All rats were treated by gavage administration for two weeks consecutively.

### Behavioral measurements

Neurological evaluation was performed by assessing the neurological deficit score^33,34^ 24 h after modeling and every week, with each point characterized by the following attributes: 0 = no symptoms of neurological dysfunction; 1 = failure to extend left forelimb fully; 2 = circling to the contralateral side; 3 = falling to the left; 4 = no spontaneous walk or in a comatose state; 5 = death.

### Infarct volume and area measurement

Rats were sacrificed by deeply anaesthetized with 5% isoflurane and euthanized by cervical dislocation. Brain tissues were kept at −20 °C for 30 min after ice cold saline washing. Coronal sections (3 mm) of the frozen brains were cut with the sharp blades and stained with 2% 2, 3, 5-triphenyltetrazolium chloride (TTC, dissolved in PBS solution, pH 7.4) at 37 °C for 30 min in the dark. Sections were reversed every 5 min to keep uniform coloring of the sections. Viable tissues were stained in deep red while the infarcts remain unstained. TTC-stained sections were photographed with a digital camera after being fixed with 4% paraformaldehyde. Finally, images were captured by a Nikon D700 digital camera (Nikon, Japan).

The infarct volume of each section was calculated using Image-Pro Plus 6.0 image analysis system (Media Cybernetics, USA). The infarct area (IS) was calculated as previously described using the following equation^35^: IS = (1 − S_1_/S_2_)*100% (S_1_: ipsilateral hemisphere non-infarcted area; S_2_: contralateral hemispher area)

### Hematoxylin-Eosin (H-E) staining

After rats being sacrificed by isoflurane, the brains of rats were taken out and fixed in 4% (w/v) paraformaldehyde for 3 days. The brain block was embedded in paraffin and cut into 3 mm coronal sections. Sequentially, the sections were stained with hematoxylin and eosin (HE), dehydrated, vitrified and mounted. The program of staining was observed under the IX51 microscope (Olympus Corporation, Japan).

### Content measurement of SOD, CAT, GSH-Px, LPO and MDA

Cells or serum of rats was collected for the content determination according to the manufacturer’s protocols. The optical density (OD) of samples was obtained with microplate reader (Thermo Scientific, MA, USA) at wavelength of 550 nm (SOD), 405 nm (CAT), 412 nm (GSH-Px), 586 nm (LPO) and 532 nm (MDA) respectively.

### Enzyme-linked immunosorbent assay (ELISA) for MMP-9, PA and PAI-1

The levels of MMP-9, PA and PAI-1 in serum of MCAO rats were determined with ELISA according to manufacturer’s instructions. The samples were readed at 450 nm using a microplate reader (Thermo Scientific, MA, USA). The contents of MMP-9, PA and PAI-1 were calculated according to the standard curve.

### Immunohistochemistry

Brain tissues were removed and immediately immersed in formaldehyde solution. Paraffin embedded tissues were sectioned (5 μm thick) and labeled with 3% H_2_O_2_, 3% normal goat serum. Briefly, sections were incubated with anti-mouse antibodies overnight. Then secondary antibodies, secondary biotinylated conjugates and diaminobenzidine were added as the instructions of DAB kit. Finally, slides were dehydrated, cleared, and mounted for visualization using IX51 microscope (Olympus Corporation, Japan).

### Transmission electron microscope (TEM)

Preparation of samples was performed according to previous method^36^. Sections of 1 mm3 were taken out from hippocampal CA1 region of rats’ brain and fixed with 5% glutaraldehyde followed by PBS (pH = 7.4) washing. Then sections were postfixed in 1% osmic acid and dyed with 2% uranyl acetate. Sequentially, sections were dehydrated by 50%, 70%, 90% and 100% acetone and embedded. Samples were analyzed using the JEM-1200EX transmission electron microscope (JEOL, Japan) at a voltage of 80 kV.

### Western blotting analysis

Protein levels in brain tissues were determined by Western blotting analysis. Briefly, proteins were extracted from tissue homogenates and an equal amount of total protein was separated by 10% sodium dodecyl sulfate-polyacrylamide gel electrophoresis (SDS-PAGE) and then transferred from the SDS-PAGE gel to PVDF membrane (Millipore, USA). After being blocked with 5% BSA in tris-buffered saline Tween-20 (TBST), membranes were incubated with the primary antibodies at a dilution of 1:1000 overnight. Subsequently, membrane was washed (three times, 5 min), and incubated with secondary antibody (1:5000) at 37 °C for 30 min. The blots were visualized with ECL-Plus reagent (Santa Cruz, USA) and analyzed with Image pro plus (IPP 6.0, Media Cybernetics, MD, USA) software. β-actin was used as loading control.

### Quantitative PCR (Q-PCR)

Total RNA of all brain samples were extracted by TRIzol reagent. Then it was reverse transcribed with a SuperScript III First-Strand Synthesis System for quantitative real-time polymerase chain reaction (q-PCR) following manufacturer’s indications. The Primer sequences used in this study were shown in Table 7. SYBR green of 10 µL, 6 µL molecular grade water, 1 µL of each forward and reverse primers and 2 µL cDNA were contained in each reaction of the PCR plate. The amplification was performed under the following conditions: 10 minutes at 95 °C, 50 cycles at 95 °C for 15 seconds and 60 °C for 60 seconds. Q-PCR was performed under standard conditions and all experiments were run in triplicate. GAPDH was used as the internal reference.

**Table 7:** Sequences of primers used for mRNA detection.

Indicator	Primer sequences (5′-3′)	
GAPDH	Forward	GGCCTTCCGTGTTCCTACC
	Reverse	CGCCTGCTTCACCACCTTC
Bax	Forward	CAAGAAGCTGAGCGAGTGTC
	Reverse	ACGTCAGCAATCATCCTCTG
Bcl-2	Forward	GAGTACCTGAACCGGCATCT
	Reverse	GAAATCAAACAGAGGTCGCA
Caspase-3	Forward	GCGTAAGGAAAGGAGAGGTG
	Reverse	ACAGACCAGTGCTCACAAGG

### TUNEL staining

Cell apoptosis in the brain was detected by TUNEL staining according to the manufacturer’s protocol (KeyGEN, Nanjing, China). Briefly, 5 μm slide sections were deparaffinized in Histoclear, rehydrated and treated with NaCl (0.85% w/v) for 5 min, then washed with 4% w/v paraformaldehyde and permeabilized with Proteinase K. After that sections were equilibrated and incubated with Biotinylated Nucleotide mix and recombinant Terminal Deoxynucleotidyl Transferase (rTdT) at 37 °C for 1 h till the reaction was stopped. Then endogenous peroxidases blocked with H_2_O_2_. Sections were incubated with Streptavidin-horseradish peroxidase conjugate solution and colored with a diaminobenzidine substrate. Sections were processed in parallel without rTdT as controls. Each section was visualized with light microscopy and images captured digitally and analyzed.

### Statistical analysis

All data were expressed as means ± standard deviation (SD). Statistical analysis was performed by SPSS 16.0 with one-way analysis of variance (ANOVA) based on Student’s two-tailed unpaired t-test or Dunnett’s multiple comparisons test. The value of *P* less than 0.05 was considered to be a statistically significant difference. Immunohistochemistry images and WB images were analyzed by Image-Pro Plus 6.0 (Media Cybernetics, MD, USA), a software specializes in image processing.

### Chemical compounds studied in this article

Protocatechuic (PubChem CID: 72); Oxypaeoniflorin (PubChem CID: 46882883); Catechin (PubChem CID: 73160); Albiflorin (PubChem CID: 51346141); Caffeic acid (PubChem CID: 689043); Paeoniflorin (PubChem CID: 75124163); Ferulic acid (PubChem CID: 445858); Benzoyl paeoniflorin (PubChem CID: 73157327).

## Supplementary information

Supplementary information.

## References

[1] Chen WJ (2016). Pretreatment of rats with increased bioavailable berberine attenuates cerebral ischemia-reperfusion injury via down regulation of adenosine-5′ monophosphate kinase activity. Eur. J. Pharmacol..

[2] Hart RG, Catanese L, Perera KS, Ntaios G, Connolly SJ (2017). Embolic Stroke of Undetermined Source: A Systematic Review and Clinical Update. Stroke.

[3] Litwinowicz, R. *et al*. Reduction in risk of stroke and bleeding after left atrial appendage closure with LARIAT device in patients with increased risk of stroke and bleeding: Long term results. *Catheter Cardiovasc Interv*, 10.1002/ccd.28187 (2019). 10.1002/ccd.28187 30884101

[4] Agayeva N, Topcuoglu MA, Arsava EM (2016). The Interplay between Stroke Severity, Antiplatelet Use, and Aspirin Resistance in Ischemic Stroke. J. Stroke Cerebrovasc. Dis..

[5] Yu ZH (2016). PI3K/Akt pathway contributes to neuroprotective effect of Tongxinluo against focal cerebral ischemia and reperfusion injury in rats. J. Ethnopharmacol..

[6] Wang CH (2016). Pharmacokinetics of 21 active components in Focal cerebral ischemia rats after oral administration of the active fraction of Xiao-Xu-Ming decoction. J. Pharm. Biomed. Anal..

[7] Zhang J (2018). Paeoniflorin influences breast cancer cell proliferation and invasion via inhibition of the Notch-1 signaling pathway. Mol. Med. Rep..

[8] Sun K, Fan JY, Han JY (2015). Ameliorating effects of traditional Chinese medicine preparation, Chinese materia medica and active compounds on ischemia/reperfusion induced cerebral microcirculatory disturbances and neuron damage. Acta Pharm. Sin. B.

[9] Zhai TH (2016). Unique immunomodulatory effect of paeoniflorin on type I and II macrophages activities. J. Pharmacol. Sci..

[10] Wan Y (2018). Adjuvant rhubarb alleviates organs dysfunction and inhibits inflammation in heat stroke. Exp. Ther. Med. Aug.

[11] Chen A (2017). Paeoniflorin exerts neuroprotective effects against glutamate-induced PC12 cellular cytotoxicity by inhibiting apoptosis. Int. J. Mol. Med..

[12] Ko CH, Huang CP, Lin YW, Hsieh CL (2018). Paeoniflorin has anti-inflammation and neurogenesis functions through nicotinic acetylcholine receptors in cerebral ischemia-reperfusion injury rats. Iran. J. Basic. Med. Sci..

[13] The State Pharmacopoeia Commission of People’s Republic of China, Pharmacopoeia of People’s Republic of China. Chemical Industry Press. Beijing. **1**, 147 (2015).

[14] Gu JF (2016). Combination of Ligusticum chuanxiong and Radix Paeoniae ameliorate focal cerebral ischemic in MCAO rats via endoplasmic reticulum stress-dependent apoptotic signaling pathway. J. Ethnopharmacol..

[15] Chen Z (2018). A systematic review on the rhizome of *Ligusticum chuanxiong* Hort. (Chuanxiong). Food Chem. Toxicol..

[16] Sun LJ (2018). Neuroprotective effect of total glycosides from paeonies against neurotoxicity induced by strychnos alkaloids related to recovering the levels of neurotransmitters and neuroendocrine hormones in rat serum and brain. RSC Adv..

[17] Long J (2012). Cardioprotective effect of total paeony glycosides against isoprenaline-induced myocardial ischemia in rats. Phytomedicine.

[18] Peisker T, Koznar B, Stetkarova I, Widimsky P (2017). Acute stroke therapy: A review. Trends Cardiovasc. Med..

[19] Wang PQ (2018). Pure mechanistic analysis of additive neuroprotective effects between baicalin and jasminoidin in ischemic stroke mice. Acta Pharmacol. Sin..

[20] Imai T (2016). Nrf2 activator ameliorates hemorrhagic transformation in focal cerebral ischemia under warfarin anticoagulation. Neurobiol. Dis..

[21] Hao FL (2019). The neurovascular protective effect of alogliptin in murine MCAO model and brain endothelial cells. Biomed. Pharmacother..

[22] Underly RG (2017). Pericytes as Inducers of Rapid, Matrix Metalloproteinase-9-Dependent Capillary Damage during Ischemia. J. Neurosci..

[23] Hoseini SM (2015). Evaluation of plasma MMP-8, MMP-9 and TIMP-1 identifies candidate cardiometabolic risk marker in metabolic syndrome: results from double-blinded nested case-control study. Metab..

[24] Zheng Z (2018). Tissue inhibitor of the metalloproteinases-3 gene polymorphisms and carotid plaque susceptibility in the Han Chinese population. Int. J. Neurosci..

[25] Hannocks MJ (2019). The gelatinases, MMP-2 and MMP-9, as fine tuners of neuroinflammatory processes. Matrix Biol..

[26] Bobińska K, Szemraj J, Czarny P, Gałecki P (2016). Role of MMP-2, MMP-7, MMP-9 and TIMP-2 in the development of recurrent depressive disorder. J. Affect. Disord..

[27] Qu LC, Jiao Y, Jiang ZJ, Song ZP, Peng QH (2019). Acidic Preconditioning Protects Against Ischemia-Reperfusion Lung Injury Via Inhibiting the Expression of Matrix Metalloproteinase 9. J. Surg. Res..

[28] Wu JT, Zhao D, Wu S, Wang D (2015). Ang-(1–7) exerts protective role in blood–brain barrier damage by the balance of TIMP-1/MMP-9. Eur. J. Pharmacol..

[29] Stonesifer C (2017). Stem cell therapy for abrogating stroke-induced neuroinflammation and relevant secondary cell death mechanisms. Prog. Neurobiol..

[30] Fumoto, T. *et al*. The Role of Oxidative Stress in Microvascular Disturbances after Experimental Subarachnoid Hemorrhage. *Transl Stroke Res*, 10.1007/s12975-018-0685-0 (2019). 10.1007/s12975-018-0685-0 30628008

[31] Bosoi CR, Rose CF (2013). Oxidative stress: a systemic factor implicated in the pathogenesis of hepatic encephalopathy. Metab. Brain Dis..

[32] Li WL, Fan YQ, Ji YB, Wang YP, Zhao Y (2007). Separation and purification of forulic acid and total phenolic acid from Ligusticum chuanxiong Hort by macroporous resins. Chin. J. N. Drugs.

[33] Longa EZ, Weinstein PR, Carlson S, Cummins R (1989). Reversible middle cerebral artery occlusion without craniectomy in rats. Stroke.

[34] Jiang YJ (2016). Opposing needling promotes behavior recovery and exerts neuroprotection via the cAMP/PKA/CREB signal transduction pathway in transient MCAO rats. Mol. Med. Rep..

[35] Luo D (2014). A Study on the Effect of Neurogenesis and Regulation of GSK3beta/PP2A Expression in Acupuncture Treatment of Neural Functional Damage Caused by Focal Ischemia in MCAO Rats. Evid. Based Complement. Altern. Med..

[36] Fan YH (2015). Spontaneous white matter lesion in brain of stroke-prone renovascular hypertensive rats: a study from MRI, pathology and behavior. Metab. Brain Dis..

