# Supplementary material for: The effect and mechanism of combination of total paeony glycosides and total ligustici phenolic acids against focal cerebral ischemia
Source: Sci Rep. 2020 Feb 28;10:3689. doi: 10.1038/s41598-020-60357-z (PMC7048792; doi:10.1038/s41598-020-60357-z)
Supplement: Supplementary file 1 — Supplementary information. [file 41598_2020_60357_MOESM1_ESM.pdf]

The effect and mechanism of combination of total paeony glycosides and total ligustici phenolic acids against focal cerebral ischemia

Junfei Gu [2,a], Liang Feng [1,3,a,*], Jie Song [3], Li Cui [3], Dan Liu [3], Xiaobin Jia [1*]

1. UPLC/Q-TOF-MS analysis of TPGs and TLPAs. UPLC/Q-TOF-MS analysis was performed on a Q-TOF Synapt system (Waters, Milford, MA, USA), equipped with electrospray ionization (ESI) source. Both positive and negative ion detection mode were conducted in this study. The desolvation gas (nitrogen, N_2), was set to a flow rate at 700 L/h at a temperature of 350 ℃, and the source temperature was set at 120 ℃. The capillary and cone voltages were set at 2.5 kV and 40 V, respectively. The mass data were recorded within the range of 50-1200 Da. The Q-TOF acquisition rate was set at 0.2 s with the transfer collision energy (E_C) of 4 V.

Chromatographic separations were performed on a Waters Acquity UPLC BEH C_{18} column (50 mm × 2.1 mm, 1.7 µm; Waters, Milford, MA, USA). The mobile phase system consisting of acetonitrile (A) and 0.1% formic acid in water solution (B) was applied with the flowing gradient program: 0-2 % A at 0-2 min; 2%-6 % A at 2-2.1 min; 6%-8 % A at 2.1-4.0 min; 8%-20 % A at 4.0-15.0 min; 20%-2% Aat 15.0-15.1min2%-2% A at 15.1-16.0 min. The flow rate was set at 0.2 mL/min. The injection volume of 3.0 µL was used for TPGs and TLPAs samples. The column temperature was maintained at 30 ℃. Ultraviolet (UV) absorbances were detected at 275 nm. The data was acquired and screened using the MassLynx software (Version 4.1, Waters) for the next analysis.

Component analysis of TPGs and TLPAs. Components of TPGs and TLPAs were analyzed and identified by UPLC/Q-TOF-MS. The chromatogram showed that samples could be separated satisfactorily under the UPLC conditions within 14 min. Four main components in TPGs were identified as oxypaeoniflorin, albiflorin, paeoniflorin and benzoyl paeoniflorin (Fig. **1A**). Oxypaeoniflorin, showed a series of quasi-molecular ion peak at *m/z* 519.8 $[M+Na]^+$, *m/z* 334.8 $[M+H^+\text{-glc}]^+$. Albiflorin, was identified by a series of quasi-molecular ion peak at *m/z* 503.8 $[M+Na]^+$, *m/z* 406.1 $[M+H\text{-}C_6H_4]^+$, *m/z* 362.1 $[M+H\text{-}C_6H_4\text{-}CO_2]^+$. Paeoniflorin, was displayed as a series of quasi-molecular ion peak at *m/z* 525.1 $[M+HCOOH\text{-}H]^-$, *m/z* 449.6 $[M\text{-}CH_2OH\text{-}H]^-$. Benzoyl paeoniflorin was displayed as a series of quasi-molecular ion peak at *m/z* 607.9 $[M+Na]^+$, *m/z* 449.6 $[M+H\text{-}C_6H_4]^+$. The content of TPGs was 47.92%, and the content of oxypaeoniflorin, albiflorin, paeoniflorin and benzoyl paeoniflorin was 0.41%, 12.45%, 33.67% and 1.39%, respectively.

The other four main components in TLPAs were identified as protocatechuic, catechin, caffeic acid and ferulic acid (Fig. **1B**). Protocatechuic, displayed a series of quasi-molecular ion

peak at *m/z* 108.6 $[M-H-CO_2]^-$, *m/z* 76.7 $[M-H-CO_2-O-O]^-$. Catechin, displayed a series of quasi-molecular ion peak at *m/z* 253.1 $[M-H-CO_2]^-$, *m/z* 181.7 $[M-H-O_2-C_6H_4]^-$. Caffeic acid, displayed a series of quasi-molecular ion peak at *m/z* 134.8 $[M-H-CO_2]^-$, *m/z* 118.7 $[M-H-CO_2-O]^-$. Ferulic acid, displayed a series of quasi-molecular ion peak at *m/z* 149.0 $[M-H-CO_2]^-$, *m/z* 118.7 $[M-H-CO_2-O]^-$. The content of TPGs was 41.43%, and the content of protocatechuic, catechin, caffeic acid and ferulic acid was 2.98%, 19.94%, 3.94% and 14.57%, respectively.

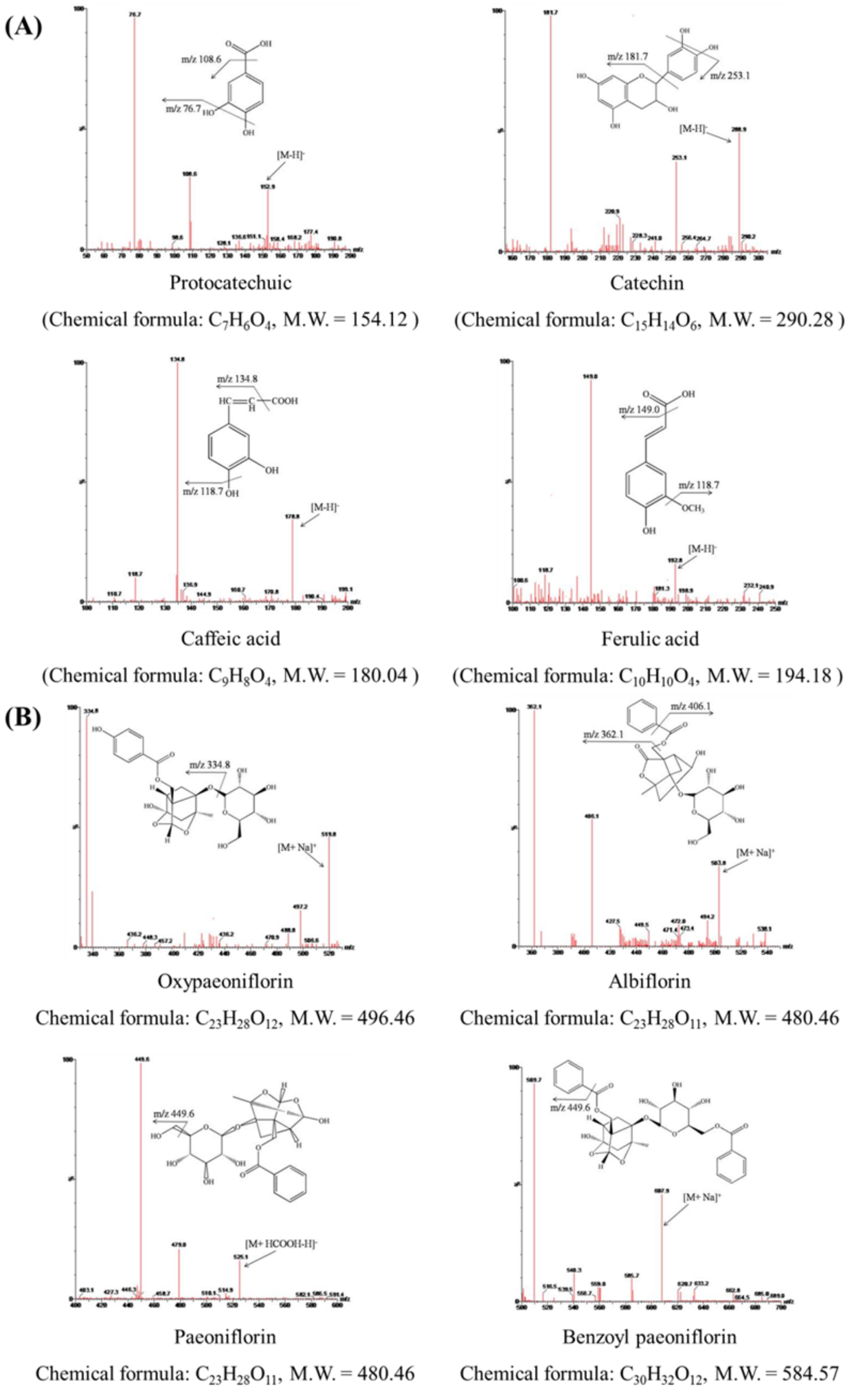

Figure 1. (A) Chemical formula of four main components in TPGs; **(B)** Chemical formula of four main components in TLPAs.

2. The original western blots figures:

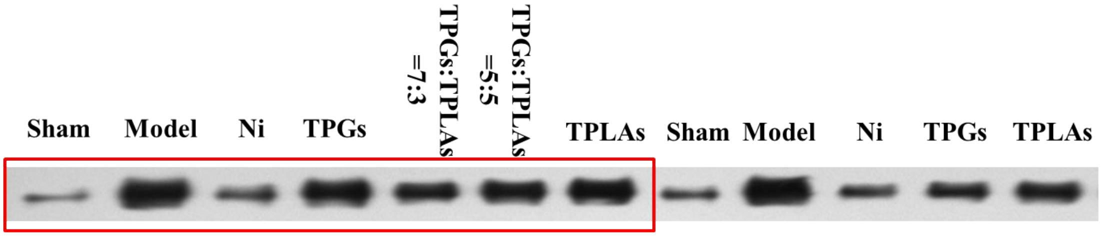

MMP-2 (72 kDa)

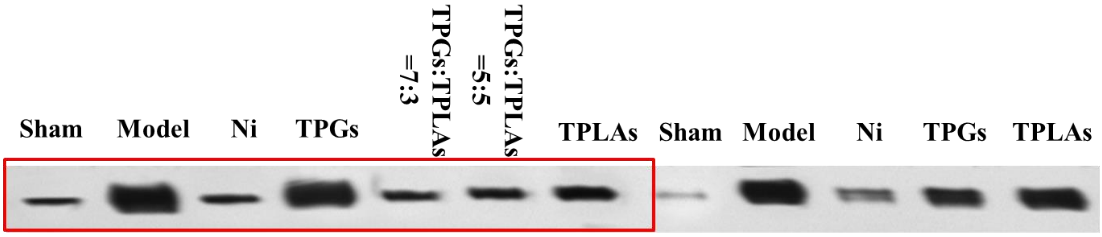

MMP-9 (92 kDa)

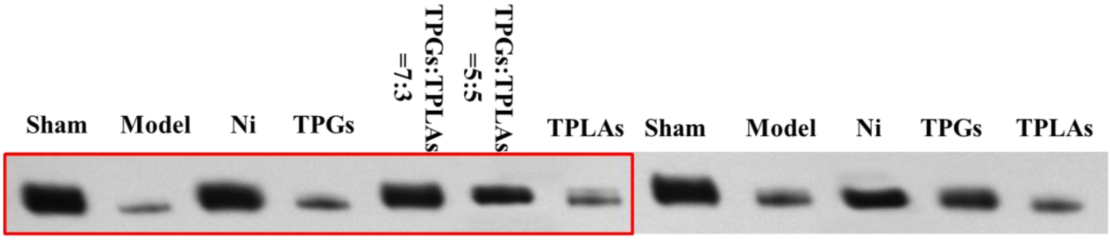

TIMP-1 (27 kDa)

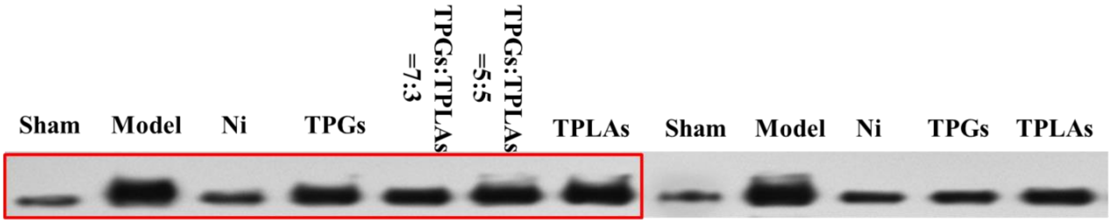

Bax (21 kDa)

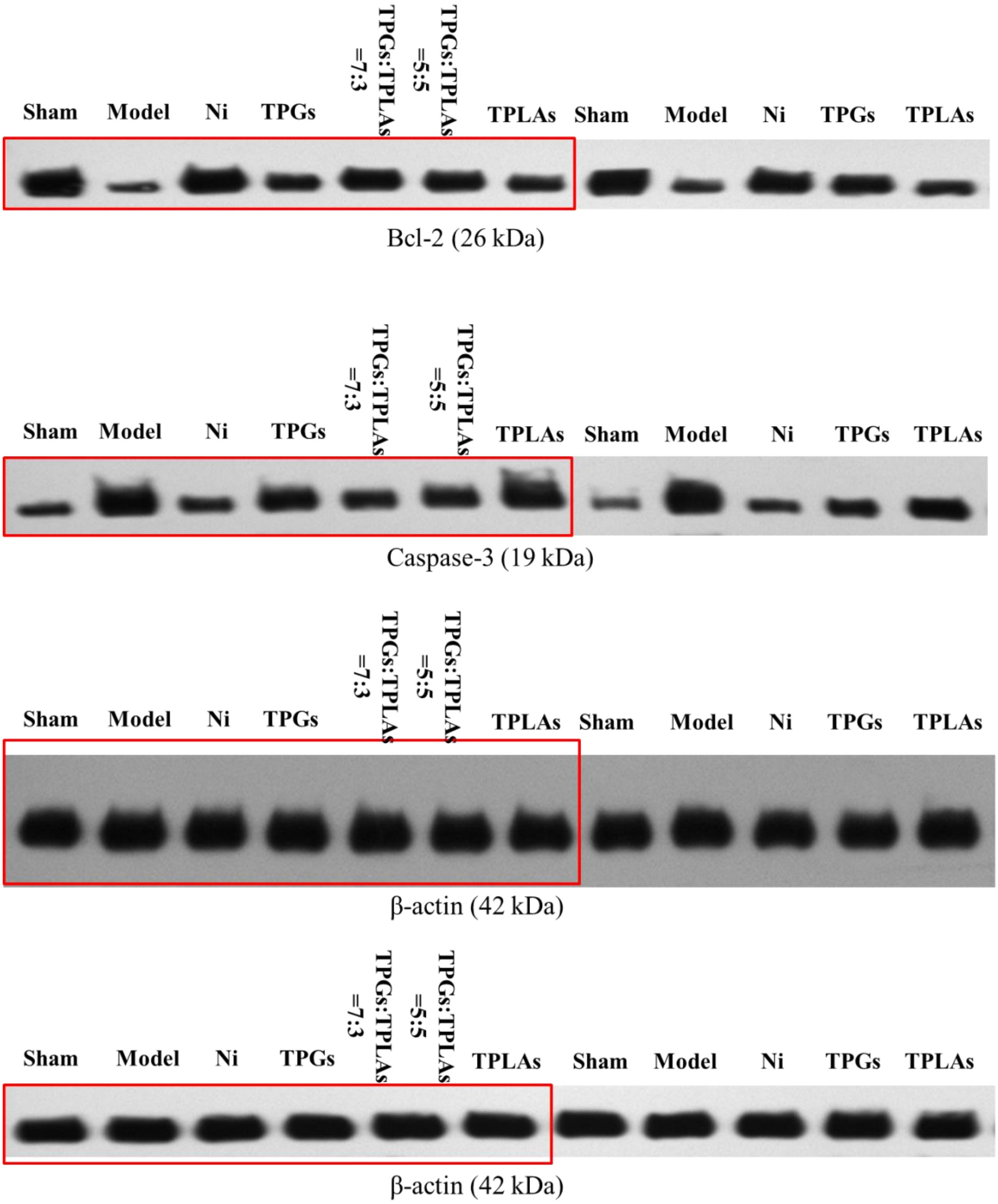
Sham
Model
Ni
TPGs
TPGs:TPLAs =7:3
TPGs:TPLAs =5:5
TPLAs
Sham
Model
Ni
TPGs
TPLAs
Bcl-2 (26 kDa)
Sham
Model
Ni
TPGs
TPGs:TPLAs =7:3
TPGs:TPLAs =5:5
TPLAs
Sham
Model
Ni
TPGs
TPLAs
Caspase-3 (19 kDa)
Sham
Model
Ni
TPGs
TPGs:TPLAs =7:3
TPGs:TPLAs =5:5
TPLAs
Sham
Model
Ni
TPGs
TPLAs
β-actin (42 kDa)
Sham
Model
Ni
TPGs
TPGs:TPLAs =7:3
TPGs:TPLAs =5:5
TPLAs
Sham
Model
Ni
TPGs
TPLAs
β-actin (42 kDa)
